# Does pelvic fixation impact reoperation outcomes for neuromuscular scoliosis surgery? A 10-year matched cohort analysis

**DOI:** 10.1007/s43390-026-01352-8

**Published:** 2026-04-09

**Authors:** Suhas K. Etigunta, Vivien Chan, Adeesya Gausper, Andy Liu, Christopher Mikhail, Alexander Tuchman, Kenneth D. Illingworth, David L. Skaggs

**Affiliations:** 1 Department of Orthopaedic Surgery, Cedars Sinai Medical Center, Los Angeles, CA USA; 2 Department of Neurosurgery, Cedars-Sinai Medical Center, Los Angeles, CA USA; 3 Alberta Children’s Hospital, University of Calgary, Calgary, Alberta Canada

**Keywords:** Neuromuscular scoliosis, Reoperation, Pelvic fixation, Posterior spinal fusion

## Abstract

**Purpose:**

To report revision surgery rates in neuromuscular scoliosis (NMS) patients undergoing posterior spinal fusion with or without pelvic fixation.

**Methods:**

The PearlDiver database identified patients who underwent posterior spinal fusion for NMS from 2015 to 2021. Two cohorts—patients with pelvic fixation and those without—were matched for age, gender, number of levels fused, and wheelchair use. Primary outcomes were rates of subsequent posterior spinal fusion and instrumentation, and spinal wound debridement. Secondary outcome assessed subsequent posterior spinal fusion rates after further matching for wound debridement status.

**Results:**

A total of 952 patients (476 with pelvic fixation, 476 without) were analyzed. The 10-year rate of subsequent posterior spinal fusion was similar between the two groups (6.9% pelvic fixation vs. 7.1% no pelvic fixation, *p* > 0.05). Subsequent posterior spinal fusion was more common in the early postoperative period (< 1 year). Wound debridement was more frequent in the pelvic fixation cohort at 10 years (10.5% vs. 6.1%, *p* < 0.05), with a higher proportion requiring early intervention within the first two months postoperatively.

**Conclusion:**

No significant difference was found in subsequent posterior spinal fusion rates between the pelvic fixation group and no pelvic fixation group. The pelvic fixation group exhibited higher rates of spinal wound debridement in the early postoperative period. Our results suggest that the addition of pelvic fixation may not be necessary in every case to reduce the rates of instrumentation revision or failure. These findings represent associations within matched cohorts and provide comparative outcome data rather than guidance for individual patient decision-making.

**Supplementary Information:**

The online version contains supplementary material available at https://doi.org/10.1007/s43390-026-01352-8.

## Introduction

Neuromuscular scoliosis (NMS) is a type of scoliosis that arises from underlying neuromuscular disorders, such as cerebral palsy, spinal muscular atrophy, and muscular dystrophy [1]. Compared to idiopathic scoliosis, NMS is typically more severe and progressive, often necessitating surgical intervention to achieve spinal balance, improve functional status, improve quality of life, and prevent further deformity [2, 3]. Posterior spinal fusion and instrumentation (PSFI) is the standard operative treatment, and pelvic fixation may be included to enhance stabilization in cases involving pelvic obliquity or severe deformity [3]. However, the benefits of pelvic fixation for neuromuscular scoliosis patients remain unclear. Some studies advocate for the use of pelvic fixation, highlighting its potential to improve the correction of pelvic obliquity and reduce the need for subsequent reoperations, including pelvic revisions [4, 5]. However, other studies have reported that pelvic fixation may not improve clinical outcomes, while being associated with increased intraoperative and postoperative risks, such as greater blood loss, prolonged surgical time, technical challenges, and a higher risk of infection [5, 6].

NMS patients face an increased risk of postoperative infections following spinal fusion, with infection rates significantly exceeding those seen in idiopathic scoliosis [7, 8]. This increased susceptibility is attributed to multiple patient- and surgery-related factors. Many NMS patients have compromised immune function, poor soft tissue integrity, incontinence, and malnutrition, all of which contribute to impaired wound healing and a greater risk of deep surgical site infections [9]. Furthermore, the presence of medical devices, such as ventriculoperitoneal shunts, has been identified as a significant risk factor for postoperative infections [10]. From a surgical perspective, extensive instrumentation, prolonged operative time, increased blood loss, and the inclusion of pelvic fixation have been associated with higher infection rates [11].

Current literature is limited by smaller sample sizes, emphasizing the need for larger analyses to further guide clinical decisions. Furthermore, NMS patients represent a clinically heterogeneous population, highlighting the importance of matched analyses when assessing outcomes. By utilizing a large national database with matched cohorts, we sought to provide evidence regarding the impact of pelvic fixation on long-term surgical outcomes. The primary aim of this study was to evaluate the need for subsequent PSFI and postoperative wound debridement after the index procedure in NMS patients with and without pelvic fixation.

## Methods

### Study design and database

A retrospective cohort study using the PearlDiver database, a comprehensive national longitudinal claims repository containing de-identified health records for more than 160 million patients across both public and private sectors, including Medicare, Medicaid, government, and commercial insurance plans, was performed. Patients who underwent PSFI for NMS were identified using Current Procedural Terminology (CPT) and International Classification of Diseases (ICD) codes.

**Fig. 1:**
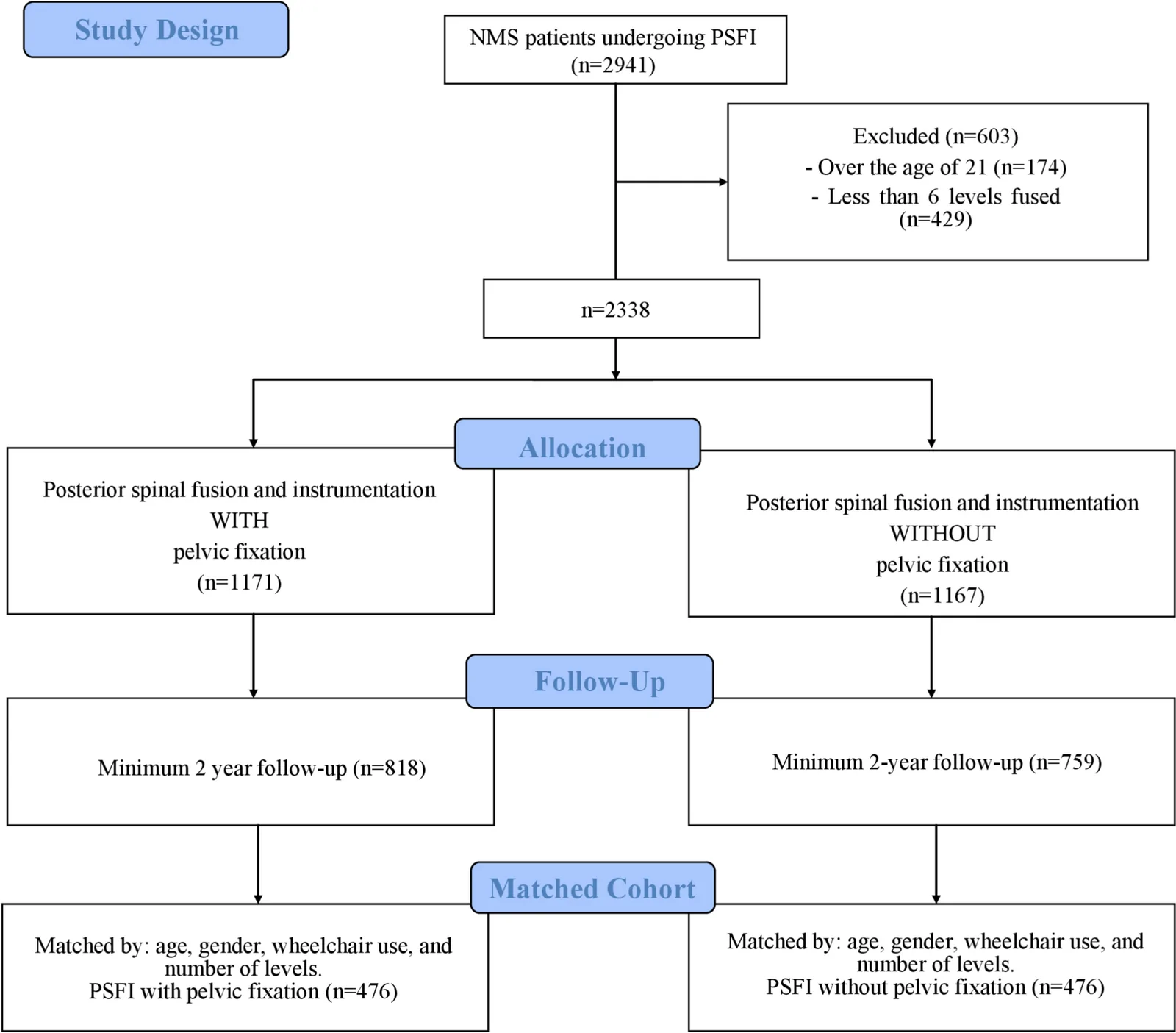
CONSORT flow diagram

Patients aged 21 years and younger who underwent PSFI for NMS between 2015 and 2021 were included in this study. The years 2015 and 2021 were only partially included. Demographic information such as age, sex, region, and year of surgery were collected. The population consisted of two cohorts: patients with pelvic fixation within 2 weeks of posterior spinal fusion and patients without pelvic fixation. This two-week period was chosen to capture staged procedures with pelvic fixation occurring on a different day than the spinal fusion due to intraoperative concerns. Patients with less than two years of follow-up after their spinal fusion surgery were excluded. Patients with fewer than 6 vertebral segments fused were also excluded. The cohorts were matched for age, gender, number of levels fused, and wheelchair use to control for potential confounding variables (Fig. 1).

The primary outcome was the rate of subsequent PSFI following posterior spinal fusion surgery at 1, 2, 3, 4, 5, and 10 years postoperatively between patients with and without pelvic fixation. PSFI within one year following the index procedure was classified as “early,” while PSFI beyond one year was “late.” The primary outcome also included subsequent wound debridement rates following the index procedure. Wound debridement in the first two months following index procedure was considered “early” intervention for infection, while wound debridement after two months was “late.” A secondary outcome looked at the need for subsequent PSFI after additional matching for spinal wound debridement status.

Data analysis was done using PearlDiver software and Python (version 3.6.5, Python Software Foundation, Beaverton, Oregon). Statistical analyses were performed using chi-square tests, with alpha value set at < 0.05.

## Results

A total of 952 patients were included in the analysis, with 476 patients undergoing PSFI with pelvic fixation and 476 without pelvic fixation (Table 1); 52.9% were female and 47.1% were male. Regional representation was distributed across the Northeast (24.3%), South (23.0%), Midwest (38.8%), and West (12.9%). Surgeries occurred between 2015 and 2021, with 62.4% of patients aged 10 to 14 years at the time of surgery.

**Table 1:** Patient demographic characteristics

Sex	*N* = 952
Female	504 (52.9%)
Male	448 (47.1%)
Region	
Northeast	231 (24.3%)
South	219 (23.0%)
Midwest	369 (38.8%)
West	123 (12.9%)
Unknown	10 (1.1%)
Year of surgery	
2015	28 (2.9%)
2016	173 (18.2%)
2017	150 (15.8%)
2018	157 (16.5%)
2019	225 (23.6%)
2020	170 (17.9%)
2021	49 (5.1%)
Age range	
5–9	24 (2.5%)
10–14	594 (62.4%)
15–19	314 (33.0%)
20–21	20 (2.1%)

### Subsequent PSFI

Rates for subsequent PSFI over a 10-year postoperative follow-up are presented in Table 2. No statistically significant differences were observed between the two groups at 1, 2, 3, 4, 5, or 10 years postoperatively. In patients with pelvic fixation, 4.8% underwent “early” subsequent PSFI (within 1 year of the index procedure), compared to 2.9% in those without pelvic fixation. The timing of subsequent PSFI differed within each group, with a statistically higher proportion of early PSFI (within 1 year) compared to late PSFI (between 1 and 10 years) in both the pelvic fixation cohort (69.7% early vs. late, *p* < 0.05) and the no pelvic fixation cohort (41.2% early vs. late, *p* < 0.05). At 10 years, the rates were 6.9% for the pelvic fixation group and 7.1% for the no pelvic fixation group. When isolating patients who underwent late subsequent PSFI, there was no significant difference between the no pelvic fixation cohort (4.2%) as compared to the pelvic fixation cohort (2.1%) (*p* > 0.05).

**Table 2:** Subsequent posterior instrumentation rates following posterior fusion with pelvic fixation vs. without pelvic fixation in patients with neuromuscular scoliosis

Time	Matched with pelvic fixation (*n* = 476)	%	Matched without pelvic fixation (*n* = 476)	%	*p* value
1 year	23	4.8	14	2.9	> 0.05
2 years	28	5.9	23	4.8	> 0.05
3 years	29	6.1	28	5.9	> 0.05
4 years	29	6.1	31	6.5	> 0.05
5 years	32	6.7	31	6.5	> 0.05
10 years	33	6.9	34	7.1	> 0.05
**Early (< 1 year)**	23	4.8	14	2.9	> 0.05
**Late (2–10 years)**	10	2.1	20	4.2	> 0.05

Significance was defined as *p* < 0.05

Analysis matched for age, gender, number of levels, and wheelchair use

### Subsequent wound debridement

Among patients with pelvic fixation, 5.7% underwent spinal wound debridement within the first two months (early spinal wound debridement), compared to 3.2% in those without pelvic fixation (Table 3). Rates of spinal wound debridement were significantly different after the first month (*p* < 0.05). At 10 years, the rates of spinal wound debridement for the pelvic fixation group and no pelvic fixation group were 10.5% and 6.1%, respectively.

**Table 3:** Subsequent wound debridement rates following posterior fusion with pelvic fixation vs. without pelvic fixation in patients with neuromuscular scoliosis

Time	Matched with pelvic fixation (*n* = 476)	%	Matched without pelvic fixation (*n* = 476)	%	*p* value
1 month	21	4.4	11	2.3	> 0.05
2 month	30	6.3	15	3.2	** < 0.05**
1 year	40	8.4	22	4.6	** < 0.05**
2 years	44	9.2	23	4.8	** < 0.05**
3 years	45	9.5	25	5.3	** < 0.05**
4 years	49	10.3	25	5.3	** < 0.05**
5 years	50	10.5	27	5.7	** < 0.05**
10 years	50	10.5	29	6.1	** < 0.05**
**Early (< 2 mo.)**	30	6.3	15	3.2	** < 0.05**
**Late (2 mo.–10 years)**	20	4.2	14	2.9	> 0.05

Significance was defined as *p* < 0.05

Analysis matched for age, gender, number of levels, and wheelchair use

The timing of subsequent spinal wound debridement was significantly different in both cohorts: 57.4% of patients in the pelvic fixation cohort received early debridement (within two months of the index procedure) versus late debridement (from 2 months to 10 years), compared to 42.9% in the no pelvic fixation cohort (*p* < 0.05 for both). However, when isolating patients who underwent late wound debridement (after 2 months), there is no difference in wound debridement rates between the pelvic fixation (4.2%) and no pelvic fixation cohorts (2.9%).

### Secondary analysis of subsequent PSFI matching for subsequent wound debridement

Table 4 reports PSFI rates after matching for spinal wound debridement status along with the previously matched variables in a secondary analysis. 3.7% of patients with pelvic fixation required subsequent instrumentation within the first year, compared to 3.1% of those without pelvic fixation. At 10 years, these rates were 4.8% and 6.5%, respectively. No statistically significant differences were observed between the two groups throughout the follow-up period. The timing of subsequent PSFI remained significant, with a greater proportion of patients undergoing early compared to late PSFI in both the pelvic fixation (77.3%) and no pelvic fixation (47.7%) cohorts. However, among those requiring late PSFI, the no pelvic fixation cohort (3.5%) had a significantly higher proportion of patients compared to the pelvic fixation cohort (1.1%) (*p* < 0.05).

**Table 4:** Subsequent posterior instrumentation rates following posterior fusion with pelvic fixation vs. without pelvic fixation in neuromuscular scoliosis

Time	Matched with pelvic fixation (*n* = 459)	%	Matched without pelvic fixation (*n* = 459)	%	*p* value
1 year	17	3.7	14	3.1	> 0.05
2 years	20	4.4	20	4.4	> 0.05
3 years	20	4.4	25	5.4	> 0.05
4 years	20	4.4	26	5.7	> 0.05
5 years	22	4.8	26	5.7	> 0.05
10 years	22	4.8	30	6.5	> 0.05
**Early (< 1 year)**	17	3.7	14	3.1	> 0.05
**Late (2–10 years)**	5	1.1	16	3.5	** < 0.05**

Significance was defined as *p* < 0.05

Analysis matched for age, gender, number of levels, wound debridement status, and wheelchair use

## Discussion

The primary finding from this study was that when matched for age, gender, number of levels, and wheelchair use, no significant differences in need for subsequent PSFI were observed between groups, with rates at 10 years being 6.9% for the pelvic fixation group and 7.1% for the no pelvic fixation group. Significance was defined as *p* < 0.05. However, spinal wound debridement was significantly higher in the pelvic fixation group at 2 months postoperatively, with rates of 10.5% compared to 6.1% in the non-pelvic fixation group at 10 years. This study represents the largest investigation of patients undergoing PSFI for NMS, offering insights into long-term outcomes of pelvic fixation in PSFI. To the author’s knowledge, it is also the first database study to query ICD coding of wheelchair use as a proxy to evaluate and match for the ambulatory status of patients.

Evidence in current literature remains inconsistent regarding the clinical, radiographic, and reoperation outcomes associated with the inclusion of pelvic fixation in PSFI for patients with NMS. In a multicenter study, Nielsen et al. found that pelvic fusion during the index surgery resulted in a significantly lower reoperation rate (22.9% vs. 50.05%) in a cohort of 285 neuromuscular scoliosis patients, while achieving similar pelvic obliquity correction [12]. However, the revision surgery group in their study consisted of only 14 patients, limited by the low incidence of revision pelvic fusion and small sample size. Tøndevold et al., in a retrospective cohort study of 91 nonambulatory patients with NMS, recommended incorporating pelvic fixation for all patients with coronal or sagittal imbalance. They found that pelvic fixation resulted in improved correction of the primary curve and pelvic obliquity without significant differences in operative time, blood loss, or complication rates [4].

Conversely, other studies have reported no differences in clinical and radiographic outcomes for NMS patients receiving PSFI with and without pelvic fixation. Yang et al. reported no significant differences in scoliosis or pelvic obliquity correction, loss of correction, or complication rates among patients treated with pelvic fixation, fixation to S1, or fixation to L5 [6]. Baymurat et al. further corroborated these findings, demonstrating comparable radiographic correction outcomes with or without pelvic fixation [13]. Farshad et al. found no significant differences in complication or revision rates between patients with or without pelvic fixation, concluding that pelvic fixation did not improve mobility outcomes in wheelchair-bound patients with mild pelvic obliquity and larger Cobb angles [14]. They reported a 28.6% overall revision rate, most commonly due to screw loosening, infection, or implant failure.

The present study assessed subsequent PSFI rates, finding no difference between pelvic fixation and no pelvic fixation groups over 10 years. Higher rates of reoperation were identified within the first year postoperatively, which may reflect reoperation due to reasons besides curve progression, such as infection, surgical complications, or instrument-related complications [11, 15].

Notably, wound debridement rates were significantly higher in the early postoperative period as compared with the late postoperative period in both the pelvic fixation (57.4% early) and no pelvic fixation cohorts (42.9% early). Furthermore, isolating late wound debridement (after two months) shows no significant difference in wound debridement rates between the two cohorts. This finding suggests that the increased risk of wound debridement in the pelvic fixation cohort is confined to the early postoperative period, after which the risk becomes comparable between the two groups. Pelvic fixation procedures may inherently require greater soft-tissue dissection and longer operative times, both of which are established risk factors for early postoperative infection. Because operative time and intraoperative blood loss are not available in the PearlDiver dataset, it is possible that the higher rate of early wound debridement observed in the pelvic fixation cohort reflects, at least in part, the increased technical demands and tissue exposure associated with these more extensive constructs.

In a secondary analysis, patients were matched for spinal wound debridement status to assess whether receiving subsequent spinal wound debridement was impacting subsequent posterior instrumentation rates. With this further matched cohort, there were still no significant differences in subsequent posterior instrumentation rates between pelvic fixation and no pelvic fixation cohorts. Notably, the timing of subsequent PSFI remained significant, with a higher proportion of patients undergoing early rather than late PSFI in both cohorts. However, among those requiring late PSFI, the no pelvic fixation cohort had a significantly higher proportion of patients compared to the pelvic fixation cohort. This finding suggests that while the risk of early reoperation is comparable between groups, late reoperations may be driven by factors such as curve progression or mechanical failure, which pelvic fixation may help mitigate. By providing enhanced distal stability, pelvic fixation may reduce the likelihood of progressive deformity that necessitates later surgical revision [12].

This study has several limitations that warrant consideration. First, the retrospective design and reliance on administrative claims data introduce the potential for coding inaccuracies, which could affect the identification of procedures and complications. Furthermore, the database lacks detailed clinical information such as curve size, pelvic obliquity, and patient-reported outcomes. These factors are key determinants in the decision to perform pelvic fixation and are not captured in the dataset, introducing the potential for confounding by indication, as patients undergoing pelvic fixation may represent a more severe subset. Importantly, the large sample size offsets the constraints in data granularity. While the use of matched cohorts mitigates some bias, residual confounding factors, such as severity of deformity or surgeon-specific preferences, may still influence the results. The study’s reliance on ICD coding for wheelchair use as a proxy for ambulatory status, while novel, may not fully capture variations in patient mobility or functional independence. The lack of radiographic data limits our ability to assess the impact of pelvic fixation on deformity correction and maintenance over time. Moreover, the decision for reoperation cannot be interpreted as a purely objective measure of deformity progression or mechanical failure. Instead, it represents a pragmatic, clinically meaningful endpoint reflecting the need for surgical revision as captured in national claims data. Finally, while the database includes a large and geographically diverse cohort, it is restricted to patients with insurance coverage, potentially limiting generalizability to uninsured or underinsured populations. Future research incorporating radiographic and patient-reported outcome measures is needed to provide a more comprehensive understanding of the role of pelvic fixation in neuromuscular scoliosis.

## Conclusion

This study found no significant differences in subsequent PSFI rates between patients with and without pelvic fixation when matched for age, gender, and number of levels. However, the pelvic fixation group exhibited higher rates of spinal wound debridement in the early postoperative period. Secondary analysis with additional matching for wound debridement status did not influence subsequent PSFI rates. These findings should be interpreted in the context of database limitations, as radiographic parameters such as curve severity, pelvic obliquity, and global alignment—key factors influencing the decision to perform pelvic fixation—were not available. As a result, some degree of confounding by indication may be present. Within these constraints, this study provides comparative data on reoperation and wound complication patterns in NMS patients undergoing posterior spinal fusion with and without pelvic fixation, without establishing causality or defining indications for pelvic fixation in individual patients.

## Supplementary Information

Below is the link to the electronic supplementary material.

Supplementary file1 (DOCX 12 kb)

## Data Availability

The data are available from the corresponding author upon reasonable request.
