# Supplementary material for: Does pelvic fixation impact reoperation outcomes for neuromuscular scoliosis surgery? A 10-year matched cohort analysis
Source: Spine Deform. 2026 Apr 9;14(5):1863–9. doi: 10.1007/s43390-026-01352-8 (PMC13553698; doi:10.1007/s43390-026-01352-8)
Supplement: Supplementary file 1 [file 43390_2026_1352_MOESM1_ESM.docx]

Supplemental Table 1. Dictionary of ICD and CPT codes by term.

| Neuromuscular scoliosis | ICD-10: M41.40, M41.41, M41.42, M41.43, M41.44, M41.45, M41.46, M41.47 |
| --- | --- |
| Number of levels | Less than 6 - CPT: 22800  7 to 12 levels - CPT: 22802  More than 12 levels - CPT: 22804 |
| Wheelchair Use | ICD-10: Z99.3 |
| Posterior spinal fusion | CPT: 22800, 22802, 22804 |
| Pelvic fixation | CPT: 22848 |
| Subsequent spinal wound debridement | CPT: 22010, 22015, 11042, 11043, 11044 |
| Subsequent posterior spinal fixation and instrumentation (PSFI) | CPT: 22800, 22802, 22804, 22830, 22849, 22850, 22852 |
